# Micro-CT imaging of Thiel-embalmed and iodine-stained human temporal bone for 3D modeling

**DOI:** 10.1186/s40463-021-00522-0

**Published:** 2021-06-02

**Authors:** Sebastian Halm, David Haberthür, Elisabeth Eppler, Valentin Djonov, Andreas Arnold

**Affiliations:** 1 Institute of Anatomy, University of Bern, Baltzerstrasse 2, CH-3012 Bern, Switzerland; 2 University of Bern, Hochschulstrasse 6, CH-3012 Bern, Switzerland; 3 Department of Ear Nose Throat, Spital Münsingen, Krankenhausweg 18/20, CH-3110 Münsingen, Switzerland

**Keywords:** 3D model, Iodine-staining, Thiel embalming, Human temporal bone, Micro-CT

## Abstract

**Introduction:**

This pilot study explores whether a human Thiel-embalmed temporal bone is suitable for generating an accurate and complete data set with micro-computed tomography (micro-CT) and whether solid iodine-staining improves visualization and facilitates segmentation of middle ear structures.

**Methods:**

A temporal bone was used to verify the accuracy of the imaging by first digitally measuring the stapes on the tomography images and then physically under the microscope after removal from the temporal bone. All measurements were compared with literature values.

The contralateral temporal bone was used to evaluate segmentation and three-dimensional (3D) modeling after iodine staining and micro-CT scanning.

**Results:**

The digital and physical stapes measurements differed by 0.01–0.17 mm or 1–19%, respectively, but correlated well with the literature values. Soft tissue structures were visible in the unstained scan. However, iodine staining increased the contrast-to-noise ratio by a factor of 3.7 on average. The 3D model depicts all ossicles and soft tissue structures in detail, including the chorda tympani, which was not visible in the unstained scan.

**Conclusions:**

Micro-CT imaging of a Thiel-embalmed temporal bone accurately represented the entire anatomy. Iodine staining considerably increased the contrast of soft tissues, simplified segmentation and enabled detailed 3D modeling of the middle ear.

**Supplementary Information:**

The online version contains supplementary material available at 10.1186/s40463-021-00522-0.

## Introduction

Research and development of modern hearing loss treatment options such as middle ear implants demand for enhanced visualization and virtual 3D reconstruction of fine structures within the petrous part of the temporal bone. To achieve high resolution and high precision morphologic data while preserving the integrity of the tissue, micro-computed tomography (micro-CT) imaging is a widely used method [1–5]. While spatial resolution in the small micrometer to large nanometer range is possible by micro-CT, a drawback is the low visibility of soft tissue due to its weak absorption of x-rays. To improve the radiographic visibility and enable precise segmentation of these tissues, the use of a contrast agent may be applied. Iodine solutions, osmium tetroxide and phosphotungstic acid (PTA) are the most commonly used micro-CT staining agents [6–9]. PTA diffusion successfully enabled high resolution visualization of renal soft tissue by micro-CT [10]. However, most recently, iodine staining was found more efficient compared to PTA for myocardial staining [11]. Similarly, comparing iodine in ethanol, iodine potassium iodide in water (IKI), PTA in ethanol, and nonionic iodinated contrast agent in water, 3% IKI staining was found best to enhance images contrast for 3D segmentation of oval squid brain [12]. Iodine has been reported to significantly improve the contrast-to-noise ratio of submillimeter middle ear connective tissue structures [6]. Although in a study applying IKI solution on formalin-fixated tissue, tissue shrinkage was not reported, however, due to possible tissue shrinkage, care should be taken when using them for morphological work [6, 8, 13]. Therefore, Boyde et al. successfully applied solid iodine using vapor staining [14].

In general, using fixated tissue is advantageous for research, both in terms of infectivity and maintenance of tissue for experimentation over a longer period of time.

Thiel-embalming is a so-called soft embalming method whose widespread use began in Europe [15, 16]. Thiel-solution is mainly composed of salts, chlorocresol, ethylenglycol and small amounts of formalin, preserving the natural colors of the tissue and above all, maintaining tissue suppleness, which is necessary for physiologic measurements (e.g., middle ear mechanics) [15]. For instance, Thiel-embalmed tissue has shown to be suitable for investigating middle ear micromechanics using laser Doppler vibrometry (LDV) [17].

For these reasons, Thiel embalming was employed in this pilot study in combination with solid-iodine staining and micro-CT imaging to create a detailed and precise 3D model of a human middle. This pilot aimed at four main purposes:

- To determine whether Thiel-embalmed human temporal bone is suitable for micro-CT imaging.
- To evaluate the feasibility and contrast enhancement of solid-iodine staining to improve depiction of fine connective tissue structures within the tympanic cavity of a Thiel-embalmed specimen.
- To determine whether micro-CT data of a Thiel-embalmed human temporal bone can be used to generate an accurate and detailed 3D model of the human middle ear including delicate structures.
- To verify the accuracy of this 3D model by measuring the stapes digitally and physically in different dimensions.

### Usage of Thiel-embalmed specimens

The 82-year-old male body donor was embalmed according to the Thiel method [15]. In brief, 14 l of injection solution, containing boric acid, ethylene glycol, ammonium nitrate, potassium nitrate, chlorocresol, sodium sulfite and formalin, was injected within 48 h postmortem into the femoral artery. Thereafter, the fixated body was preserved in 100 l of Thiel storage solution. A detailed list of these solutions is specified in Additional File 1.

Both temporal bones of the body donor were excised (sample size 5 cm × 3 cm × 3 cm) and preserved in Thiel solution for another month.

Two weeks prior to tomographic imaging, the temporal bones were removed from the Thiel solution bath and excess liquid was allowed to evaporate. Scanning the sample in an air instead of a liquid environment enhances contrast differences in the sample.

As a first step, the left temporal bone was CT-scanned in its native state, then iodine-stained and re-scanned. The stained dataset was compared with the unstained and used to create the 3D model.

The contralateral right temporal bone was subjected to micro-CT in its native state. This data set was imported in 3D Slicer software to digitally measure the stapes dimensions [18]. The stapes was then dissected from the temporal bone and measured manually under the microscope.

The iodine stain was applied to enhance the visibility of the soft tissue structures, while both measurements of the stapes were used to investigate the accuracy of the 3D model.

#### Micro-CT imaging

Tomography scans were performed in a SkyScan 1172 micro-CT system (Bruker, Kontich, Belgium). The samples were imaged with a source voltage of 89 kV and a source current of 112 μA. The x-ray spectrum was filtered with a 0.5 mm Aluminum/0.08 mm Copper filter. We used a 2 × 2 binned camera window with two overlapping lateral acquisitions (resulting projection size: 1336 × 3968 pixel) and acquired one projection image at every 0.2° degree of rotation (total of 180°) resulting in 1018 projections. Each projection was exposed for approximately 2.6 s; three exposures were averaged into one to increase the signal-to-noise ratio. The resulting isometric voxel size (side length) was 13.27 μm.

Projections were reconstructed into a set of PNG images using NRecon (Version 1.7.0.4, Bruker) with a ring artifact correction of 16 and a beam hardening correction of 60%.

#### Sample preparation and iodine staining

**Fig. 1:**
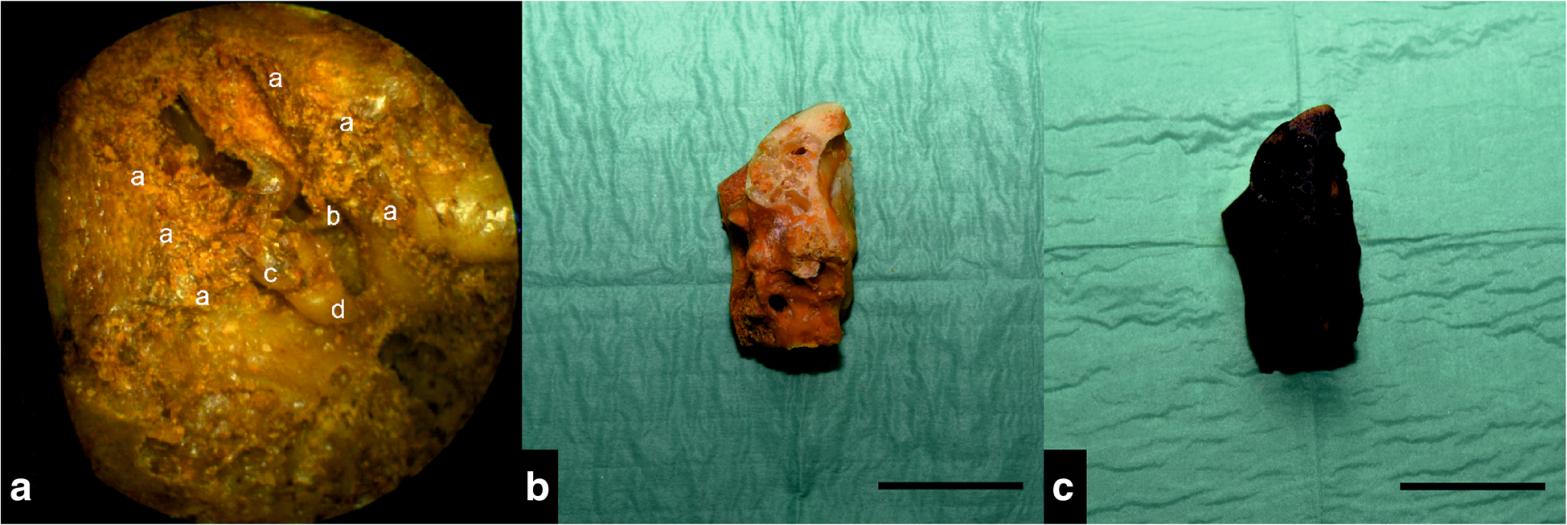
Dissection of the tympanic cavity and staining effect. **a** View into the tympanic cavity after removal of the covering mastoid cells (a). The ossicle chain is visualized: b = Stapes, c = Incus, d = Malleus. **b** Unstained and trimmed temporal bone. **c** Iodine-stained temporal bone after 3 days. Scale bar: 3 cm

Following the first CT scan, middle ear structures were iodine-stained. In order to avoid tissue shrinkage, solid iodine vapor staining was applied as described previously by Boyde et al. [14] In our study we adapted this method for complete staining of the middle ear structures. For that purpose, iodine pellets were carefully placed into the tympanic cavity after removal of some mastoid cells with Luer forceps and a preparation needle. Access and integrity of the preserved structures (malleo-incudal joint, tendons and ligaments) were verified using a flexible Atmos FESS Portable 3.8 mm otoendoscope (Fig. 1a). The temporal bone (weight 22.6 g) and 0.4 g of solid iodine were put in a closed glass jar for 72 h (Fig. 1b). After iodine evaporation, the temporal bone was dark brown in color (Fig. 1c). Subsequently, the temporal bone was left to air dry at room temperature before re-scan.

#### Unstained vs. iodine-stained images

To evaluate the effect of iodine staining, we compared the unstained and iodine-stained micro-CT data set and assessed the following five structures of interest (SOI): lateral malleal ligament, annular ligament, stapedius muscle, tensor tympani muscle and tympanic membrane.

The contrast of these SOI compared to air and bone was visually assessed and quantified by calculating the contrast to noise ratio (CNR) as follows: CNR = $\frac{mean(x)- mean(y)}{SD(y)}$ whereby x is the pixel brightness of SOI, and y is the pixel brightness of the background [19].

For each SOI, as well as the surrounding empty space, five measurement points were randomly selected and the respective CNR values measured (Additional Table 2).

We used Fiji (https://fiji.sc), an open-source platform for biological-image analysis, for image processing, calculating pixel brightness values and generating a line plot of pixel brightness [20].

As a representative for thin soft tissue structures, the tympanic membrane was selected to set a line plot of the pixel brightness over the unstained and stained tympanic membrane. To show the difference in detectability, the values were transferred to Excel (Microsoft Office 2016, Microsoft, Redmond, WA, USA) and plotted.

#### 3D model

Using Fiji, PNG files of the iodine stained micro-CT scan were converted into an .nrrd file, which was imported into 3D Slicer (Version 4.8.1, http://www.slicer.org). To expedite processing of the data in 3D Slicer, the dataset of the temporal bone was binned two-fold, resulting in images with a pixel size of 26 μm.

The middle ear structures from the dataset were manually segmented by painting the region of interest (ROI) into each third slice. The “fill between the slices” function was used to interpolate in between the manually painted slices. The segmented dataset was pasted into a label map and further processed to yield a surface model.

#### Stapes measurements: 3D model vs. removed stapes

**Fig. 2:**
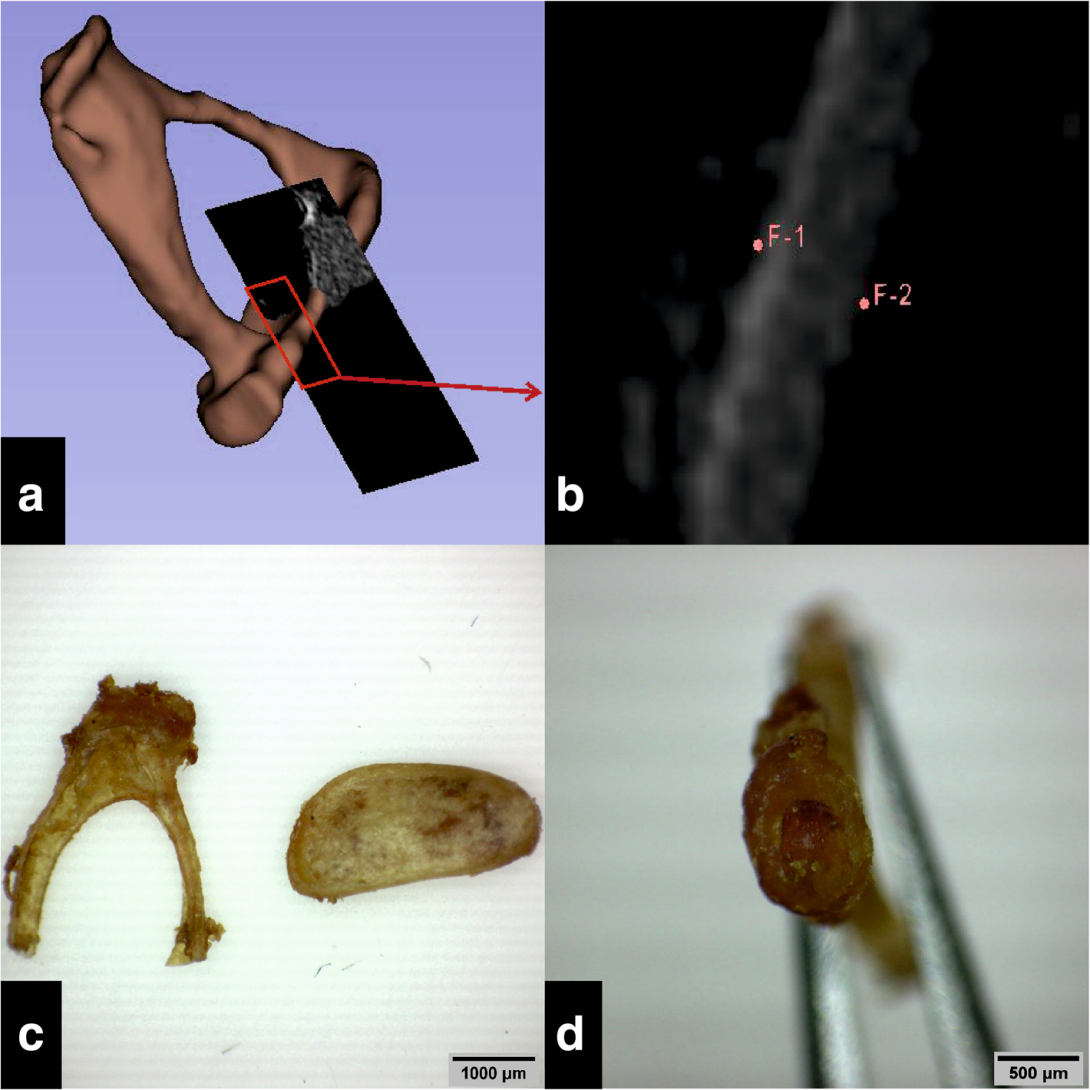
Digital (**a**, **b**) and physical (**c**, **d**) stapes measurement. **a** Simultaneous view of 2D plane and created model in 3D Slicer. **b** Zoomed 2D plane from A, placement of the measuring points (F-1, F-2). **c**, **d** Dino Capture images of the removed stapes. Stapes dimensions are indicated in Table 1

To verify whether Thiel fixation affects middle ear bones, the stapes as a representative of the ossicle was measured. The dimensions were compared with literature data. Care was taken to measure the stapes in a comparable manner to those of the published literature values (Additional Table 3). Therefore, stapes dimensions were measured digitally in 3D Slicer (Fig. 2a, b) and physically after dissection from the temporal bone (Fig. 2c, d). The dimensions were compared with each other and with literature values.

The stapes was photographed and measured with a light microscope Olympus Sz 40 (Olympus K.K., Shinjuku, Tokyo, Japan) using an AM7025X Dino-Eye Edge Eyepiece Camera (Anmo Electronics Corporation, New Taipei City, Taiwan).

Camera resolution of 2592 × 1944 pixel resulted in an image pixel size of 6 μm. Images were processed using the Dino Capture 2.0 Microscope Imaging Software (Anmo Electronics Corporation, New Taipei City, Taiwan).

## Results

### Unstained vs. iodine-stained images

**Fig. 3:**
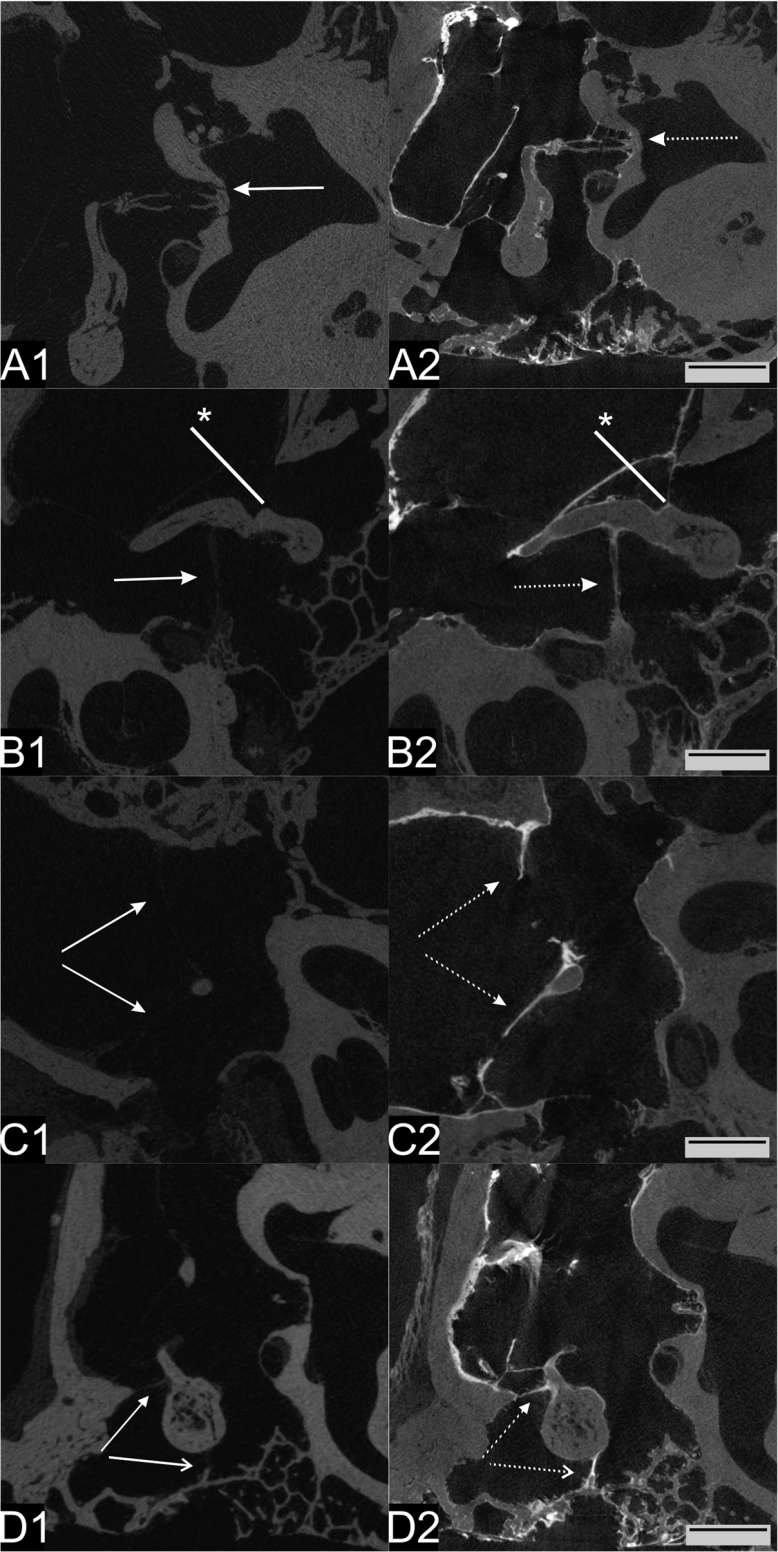
Influence of iodine staining on micro-CT imaging. The continuous arrow points to the unstained structure (1), the dotted arrow to the stained structure (2). **A** Annular ligament. Note: the ligament is better visible in the unstained sample. **B** Tensor tympani muscle. *: Region of the line plot shown in Fig. 4, approximately 3 mm long. **C** Tympanic membrane. Note: unstained area in the lower and perforated area in the upper part (probably retraction after staining). **D** Ligaments of malleus. Open arrowhead = superior malleal ligament. Filled arrowhead = lateral malleal ligament. Scale bar = 3 mm

Native micro-CT visualized the ossicle chain and almost all middle ear soft tissue structures investigated (Fig. 3. A1-D1), except for the chorda tympani.

**Fig. 4:**
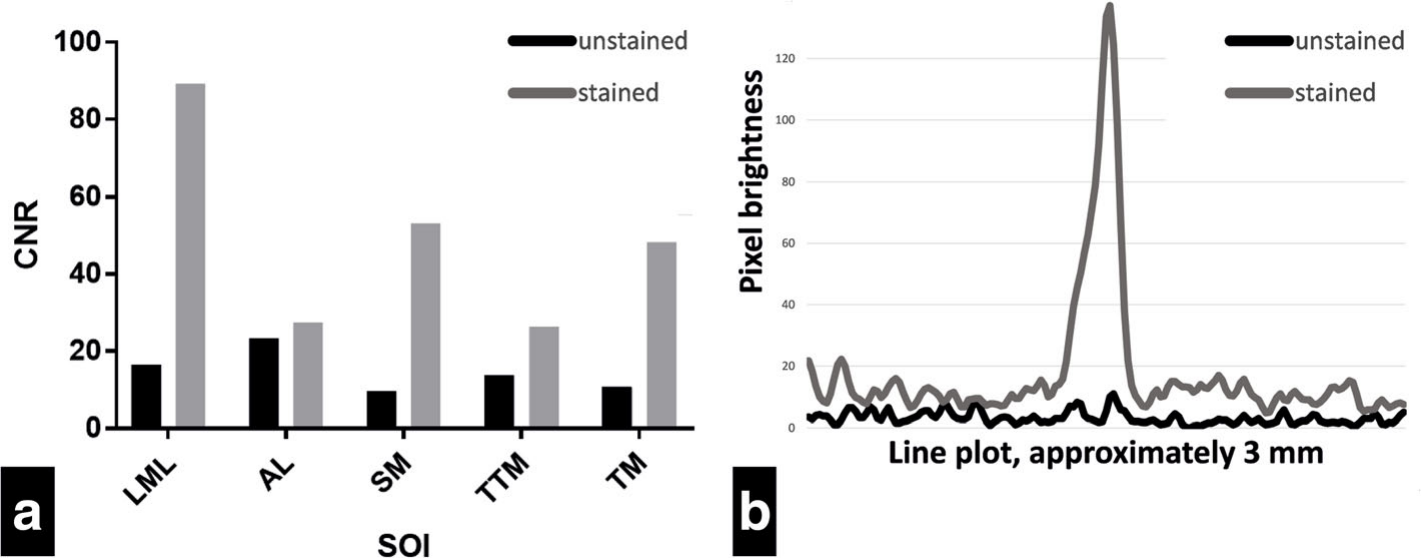
CNR of different SOI, line plot of brightness across tensor tympani muscle. **a** Contrast to noise ratio (CNR) of structures of interest (SOI), before and after staining. SOI: LML = lateral malleal ligament. AL = annular ligament. SM = stapedius muscle. TTM = tensor tympani muscle. TM = tympanic membrane. **b** Line plot of brightness through tympanic membrane over a distance of approximately 3 mm (depicted in Fig. 3b), before and after staining. Compare the visible peak in the stained plot to the not visible peak in the unstained plot. Pixel brightness = 8-bit grayscale, 0–255

However, visibility and demarcation of the SOI were pronouncedly enhanced after solid iodine-staining, due to an improved contrast of the soft tissue structures, including the tympanic membrane (Fig. 3. B2, C2). The CNR values of the SOI (detailed in Additional Table 2) were, on average, 3.7 times higher (Range: 1.17–5.59, SD: 1.83) than the CNR of the unstained soft tissues (Fig. 4a). The largest CNR increase occurred for the stapedius muscle (Δ 5.59) and the lateral malleal ligament (Δ 5.41). As depicted by the line plot, pixel brightness was substantially increased in the region of the tympanic membrane relative to the unstained tympanic membrane (Fig. 4b). Notably, while the annular ligament could be identified without iodine-staining as a gap between the stapes footplate and the adjacent promontory bone (Fig. 3. A1), after iodine staining, the annular ligament was depicted with a similar contrast as the osseous tissue directly adjacent to it (Fig. 3. A2), despite a slightly higher CNR (Additional Table 2). This made its identification after iodine-staining (Fig. 3. A2) more difficult than without iodine staining. Thus, combination of unstained and solid iodine-stained micro-CT, yielded an excellent depiction of the delicate structures of the middle ear (Fig. 3).

### 3D model

The improved visibility due to the staining has led to an improved delimitation from the background and improved assignment of middle ear soft tissue structures, except for the annular ligament as described above. This made segmentation considerably easier and more accurate. The small perforation of the tympanic membrane, visible by micro-CT (Fig. 3. C2), was manually closed during the digital segmentation procedure. It is remarkable that even the chorda tympani, which was not detectable without iodine-stain, was visible in the stained scan (data not shown) and could be segmented.

**Fig. 5:**
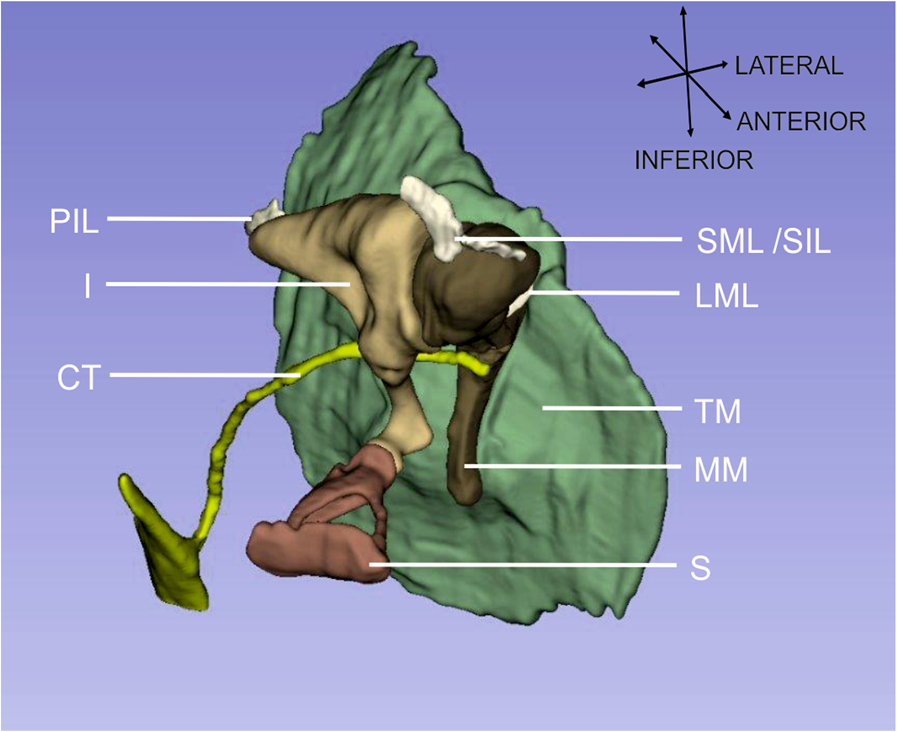
3D model. 3D model of the middle ear structures segmented in 3D Slicer. I: incus, MM: manubrium of malleus, S: stapes, CT: chorda tympani, LML: lateral malleal ligament, PIL: posterior incudal ligament, SIL: superior incudal ligament, SML: superior malleal ligament, TM: tympanic membrane. The small perforation of the tympanic membrane visible in Fig. 3. C was manually closed during the segmentation procedure

The resulting 3D model (Fig. 5) accurately depicts the 3D arrangement and position of the ossicle, as well as the detailed position, insertions and tensile direction of the associated soft tissues, including the chorda tympani.

### Stapes measurements: 3D model vs. removed stapes

Large absolute deviations (∆1) between 3D Slicer and light microscope occurred in large stapes structures and large percentage deviations (∆2) in small stapes structures (Table 1). For tiny structures, small absolute deviations already resulted in large percentage differences. In general, the measured values of both methods differed by 0.01–0.17 mm or 1–19%, respectively. Table 1 shows the digitally and physically measured stapes dimensions, literature reference values and the differences between each.

**Table 1:** Stapes dimensions and differences

Structure	Dimension [mm]			Differences		
	3D Slicer	Light microscope	Literature	∆1	∆2	∆3
length footplate	2.95	3.12	1.92 [21]–3.56 [22]	0.17	6%	
width footplate	1.44	1.47	1.16 [23]–1.4 [24]	0.03	2%	0.04–0.07
height of footplate	0.18	0.19	0.18 [21]–0.39 [21]	0.01	6%	
height of stapes	3.36	3.49	3.07 [23]–4 [24]	0.13	4%	
length of stapes head	0.98	1.04	0.85 [21]–1.49 [25]	0.06	6%	
width of stapes head	0.74	0.75	0.65 [25]–1.08 [25]	0.01	1%	
width of anterior crus	0.52	0.42	0.21 [21]–0.65 [25]	−0.1	− 19%	
width of posterior crus	0.43	0.41	0.14 [21]–0.75 [25]	−0.02	−5%	
area footplate (mm2)	3.15	3.6	2.7 [23]–3.36 [23]	0.45	14%	0.24

Table Note: Dimensions measured digitally (3D Slicer), physically (light-microscope) and from the literature (with references). ∆1 and ∆2: difference between our measuring methods in mm and % respectively. ∆3: differences to the literature values, if deviating

### Stapes measurements: 3D model & removed stapes vs literature values

The width of the footplate of 1.44 mm (3D Slicer) and 1.47 mm (light-microscope), respectively, was approximately 40 μm and 70 μm wider (∆3) than the largest reference value in the literature (Table 1).

The area of the footplate was about 0.24 mm^2^ larger than the largest reference values in the literature. Six out of nine (66%) of the physically measured values and eight out of nine (88%) of the digitally measured values were within a range of ±1 SD of the mean as compared to stapes measurement values in the literature (Additional Table 4).

## Discussion

In this pilot study, we demonstrate that a precise 3D model can be obtained from high resolution micro-CT imaging of an iodine-stained Thiel-embalmed temporal bone. The resulting images depicted all middle ear structures. Iodine vapor staining considerably facilitated segmentation of a detailed 3D model of the human middle ear, including soft tissue structures. The stapes dimensions measured in the 3D model correspond well with the physical measurement, as well as with literature values.

### Unstained vs. iodine-stained images

Micro-CT imaging of unstained Thiel-tissue provided detailed image data with well-visible middle ear structures, except the chorda tympani (Fig. 3). However, to improve the visibility and above all to visualize the trajectory of small connective tissue structures for facilitated and precise segmentation, staining is still desirable. An improved contrast enhancement and tissue depiction by iodine staining was shown by Metscher [9], while Rohani reported a quantitative improvement of the CNR [6].

By using solid iodine, Boyde and co-workers avoided possible tissue shrinkage when staining embedded blocks for histology [8, 14]. Analogous to this procedure, we used solid iodine for better contrast and placed it directly into the middle ear after removing some mastoid cells.

To the best of our knowledge, the effect of vapor iodine staining on Thiel embalmed specimen has not been reported in the literature to date. The iodine dose used in this study was empirically determined according to experiments with zebrafish (unpublished data from our institute) and based on work of Babaei [26]. We used approximately 17 mg of solid iodine per gram of sample, staining for 72 h.

For comparison, Boyde and co-workers used between 8 and 125 mg solid iodine per gram sample and achieved best results with staining up to seven days [14]. In our study, solid iodine staining of the temporal bone specimen clearly enhanced the visibility of middle ear soft tissue structures (Fig. 3), improved the CNR of the SOI by a factor up to 5.59 (mean 3.7, SD 1.83; Fig. 4a) and line plot brightness through tympanic membrane (Fig. 4b). Stapedius muscle and lateral malleal ligament (LML) showed the highest iodine uptake and therefore the largest CNR difference (Fig. 4a). Thus, iodine staining is an additional benefit for the recognition of soft tissue structures and further processing of micro-CT images.

### 3D model

Increased contrast is a notable advantage for analyzing the micro-CT image set resulting in an easier, thus quicker, but also more accurate segmentation. In contrast to our expectations, even the chorda tympani was visible and could be integrated into the 3D model (Fig. 5). As a notable exception, the annular ligament, which was only indirectly recognizable as a gap between osseous tissues, did not benefit from the iodine staining, which enhanced the contrast of the ligament to a contrast similar to the surrounding bone.

Segmentation resulted in a precise 3D model, which showed in detail the middle ear anatomy (Fig. 5).

### Stapes measurements: 3D model vs. removed stapes

The difference between both measuring methods was up to 170 μm or 19%, depending on the measured stapes structure (Table 1).

A possible explanation is that the corresponding structure had a slightly different orientation when measured digitally and physically. Furthermore, measurement differences may occur due to measuring inaccuracy related to the pixel size. A difference of 170 μm with a pixel size of 26 μm means a deviation of the measurement point of only 6.5 pixels (3D Slicer) and 28 pixels with a pixel size of 6 μm (Dino Lite camera). When a higher magnification of an image is selected to accurately set the measurement point, the individual pixels become visible. The main difficulty about setting the measurement point, is the image transition from bone to air. At high magnification, there is no clear cut-off. The border of the bony subject fades out with a gradual transition of pixels brightness from bone to air, posing a source of inaccuracy for measurement point setting (Fig. 2). For small pixel size (6 μm), there are more pixels to choose from, but the resulting deviation is small, because the pixel size is small. For a larger pixel size (26 μm), there are fewer pixels, but the resulting deviation is greater.

Table 1 shows that most of our manually measured values are minimally smaller. Probably due to the mentioned air-bone interface with a tendency to select pixels that are too bright at the transition. Setting the measurement values according to the slope of a line plot would be desirable, but is unfortunately not possible in 3D Slicer.

### Stapes measurements: 3D model & removed stapes vs literature values

Two deviating values of Table 1 must be explained. First, the manually measured width of the stapes footplate was 1.47 mm and the 3D Slicer-measured width 1.44 mm. These values moderately deviate from the 1.4 mm given in the literature [24], which, however, is only reported to one decimal place.

Secondly, the manually measured area of the stapes footplate was 3.60mm^2^, which is about 0.24mm^2^ larger than the largest reported value.

However, when calculating the area using the largest reported width and length, the resulting footplate area would be 4.11mm^2^ and consequently our measured area lies within this calculated range.

Overall, our measured values correspond well with the literature values achieved by different methods including unfixated fresh cadaver material.

We conclude that Thiel-fixation had a negligible effect on our middle ear bones and that micro-CT imaging of Thiel-tissue represents the correct anatomy. In general, digital measurement in 3D Slicer is a non-destructive method and the simultaneous viewing of the 3D model and 2D image planes allows for accurate placement of the measurement points. The disadvantage is the time-consuming creation of the image data set, with the accuracy of the 3D model depending on the time spent on segmentation.

Other methods like Orthogonal-Plane Fluorescence Optical Sectioning microscopy (OPFOS) or Synchrotron radiation phase-contrast imaging (SR-PCI) have been reported to provide high resolution imaging of the dense and soft tissue structures of the middle ear [27–33].

With the OPFOS a pixel sizes as small as 0.75 μm are reported, and with SR-PCI pixel sizes as small as 0.65 μm [28, 33]. These methods achieved excellent visualization of soft tissues and high-resolution images of small sample sizes so that a field of view of 1.64 mm × 1.38 mm in SR-PCI enabled the visualization of single, extracted middle ear bones. Expanding this view, in the present study applying iodine staining and micro-CT, a straightforward method visualizes all soft tissues of a much larger (5 × 5 × 3 cm) and intact temporal bone sample, with a relatively small voxel size of 13.27 μm.

## Limitations

In this pilot study, the sample size of two temporal bones may mean that certain confounders have not been detected. Although we do not believe in a fundamental change in the outcome of the present study, future studies applying our method may contribute further findings.

Nevertheless, Thiel embalming is time-consuming and requires many chemicals. Costs are described as 8–14 x higher than formalin fixation (300–437 Euro vs. 30–36 Euro) [16, 34]. But cost-reducing measures such as storage of several cadavers in one tank and multiple uses of a Thiel-cadaver for different clinical courses and experiments have not yet been considered.

The fixation protocol can be adapted by anatomical institutes individually. Changes of the protocol may have different impacts on the tissues and would require a re-validation of the method described here.

Furthermore, the study did not verify a possible effect of tissue shrinkage after application of solid iodine [14]. The cost for iodine is negligible with a few centimes for the 17 mg iodine we used. 3D Slicer and Fiji are both free and open-source software.

### Prospective

Thiel-fixated body donor specimens are valuable for use in postgraduate middle ear surgery [35].

Pre- and postinterventional CT-imaging may benefit from improved visibility of delicate iodine -stained structures and contribute to teach temporal bone approaches and middle ear operations. Furthermore, 3D models may demonstrate physiology, pathology and desired surgical results and reconstructions.

Precise 3D models of the ear are helpful tools in research and development of surgical ear therapies, including passive and active implants [36].

Finite element modeling (FEM) is an established method for investigation of middle ear mechanics [37]. To the best of our knowledge, no middle ear FEM has yet been created from a 3D model of a Thiel-fixed temporal bone. The resulting 3D model shall serve as basis of a dynamic middle ear FEM model. Such a model will be validated and adjusted using physiological and pathophysiological motion data from laser Doppler vibrometry (LDV) measurements of the same Thiel-embalmed temporal bone [17].

## Conclusion

In our pilot study, micro-CT imaging of a Thiel-fixated petrous bone completely and accurately depicted the anatomy, including the soft tissues.

Iodine staining considerably increased the CNR of the soft tissues, profoundly simplifying the segmentation process for generating a 3D model.

## Supplementary Information

**Additional file 1.** Composition of Thiel-embalming solutions.

**Additional file 2: Additional Table 2.** Detailed CNR calculation and pixel brightness values.

**Additional file 3: Additional Table 3.** Detailed position of the stapes measuring points.

**Additional file 4: Additional Table 4.** Detailed stapes dimensions of the literature, compared with our values.

## Data Availability

All data generated during this study are included in this published article and as additional data files. The micro-CT data are available from the corresponding author on request.
