# Supplementary material for: Micro-CT imaging of Thiel-embalmed and iodine-stained human temporal bone for 3D modeling
Source: J Otolaryngol Head Neck Surg. 2021 Jun 2;50:33. doi: 10.1186/s40463-021-00522-0 (PMC8173723; doi:10.1186/s40463-021-00522-0)
Supplement: Supplementary file 1 — Additional file 1. Composition of Thiel-embalming solutions. [file 40463_2021_522_MOESM1_ESM.docx]

**Additional File 1.** Composition of Thiel-embalming solutions. According to «Die Konservierung ganzer Leichen in natürlichen Farben» by Walther Thiel, 1992^1^

INJECTION SOLUTION

Basic solution 14.3 l, consist of:

- Water, 100 ml
- Boric acid, 3 g
- Ethylene glycol, 30 ml
- Ammonium nitrate 20 g
- Potassium nitrate, 5 g

Chlorocresol solution 0.5 l, consists of:

- Ethylene glycol 10 ml
- Chlorocresol 1 ml

Sodium sulfate 700 g

Formalin 0.3 l

IMMERSION SOLUTION

Water, 100 ml

Boric acid, 3 g

Ethylene glycol, 10 ml

Ammonium nitrate, 10 g

Potassium nitrate, 5 g

Chlorocresol solution, 2 ml (see above)

Sodium sulfite, 7 g

Formalin, 2 ml

Reference:

1. Thiel W. [The preservation of the whole corpse with natural color]. *Ann Anat.* 1992;174(3):185-195. PMID: **1503236**
